# A case report of synovial sarcoma arising in the abdominal wall muscles

**DOI:** 10.1097/MD.0000000000050484

**Published:** 2026-08-28

**Authors:** Wen-Xu Liu, Wen-Qian Liu, Ji-Guang Song, Ming-Hao Jia, Jia-Le Wang, Feng-Ling Li, Jia-You Xu

**Affiliations:** a Weifang People’s Hospital, Shandong Second Medical University, Weifang, Shandong, China; b Department of Dispensar, Shan wang Central Health Center of Linqu County, Weifang, Shandong, China.

**Keywords:** CT imaging, Fish gene testing, Immunohistochemistry, Magnetic Resonance Imaging, Mass of the abdominal wall, Synovial Sarcoma

## Abstract

**Rationale:**

Synovial sarcoma, a rare form of soft tissue sarcoma, typically manifests in the synovium of major joints. The incidence of this disease in the intermuscular region of the abdominal wall, as observed in this particular case, is an even rarer occurrence. Relying solely on conventional imaging modalities often proves insufficient for establishing a definitive diagnosis.

**Patient concerns:**

A 67-year-old female inadvertently palpated a mass in the abdomen two years ago. The mass was firm in texture and had limited mobility. The patient expressed concerns regarding the possibility of it being a malignant tumor.

**Diagnoses:**

Prior to the operation, color-Doppler ultrasonography of the local superficial mass revealed a hypoechoic area measuring 2.2 × 1.2 × 2.6 cm within the muscular layer at the site on the left abdominal wall as described by the patient. The lesion exhibited a distinct border, an irregular morphology, heterogeneous internal echoes, and a small amount of blood flow signals. postoperative pathological examination confirmed the diagnosis of synovial sarcoma.

**Interventions:**

Surgical treatment was performed, followed by 4 cycles of chemotherapy post-operation.

**Outcomes:**

Complete resection was accomplished. postoperative computed tomography (CT) reevaluation of the patient demonstrated no evidence of metastasis.

**Lessons:**

For suspicious masses in unconventional locations, systematic examinations should be conducted as soon as possible, including imaging tests and preoperative pathological biopsy, in order to reduce the rate of preoperative misdiagnosis and missed diagnoses. Early diagnosis will minimize the risks and losses for the patient.

## 1. Introduction

Synovial Sarcoma is a relatively rare and highly aggressive high-grade soft tissue sarcoma that predominantly affects adolescents and older children. It has an extremely low incidence rate of approximately 1.42 per million people.^[1]^ As a type of soft tissue sarcoma, it originates from primitive mesenchymal cells with the potential for epithelial differentiation and accounts for 5–10% of all soft tissue sarcomas.^[2]^

While synovial sarcoma can occur in any part of the body, it is most commonly found near the large joints of the extremities, with the knee being the most frequent site. Occurrences in the head and neck, chest, abdominal wall, heart, retroperitoneum, and kidney are relatively rare, with tumors arising in the abdominal wall accounting for about 5% of cases. A distinctive genomic feature of synovial sarcoma is the t(X;18) chromosomal translocation, which results in the formation of the SS18::SSX fusion oncogene and serves as a key diagnostic marker.

To date, there has been limited research on the imaging characteristics of extra-appendicular synovial sarcoma, and the number of reported cases remains small. Due to its low prevalence, lack of specific clinical symptoms, and nonspecific imaging findings, this tumor is often misdiagnosed. Most available literature consists of case reports rather than large-scale studies.

In December 2024, our department surgically resected a case of synovial sarcoma located within the abdominal wall muscles. This report aims to enhance understanding of this condition and improve preoperative diagnosis to reduce the rate of misdiagnosis.

## 2. Case information

**Figure 1.**
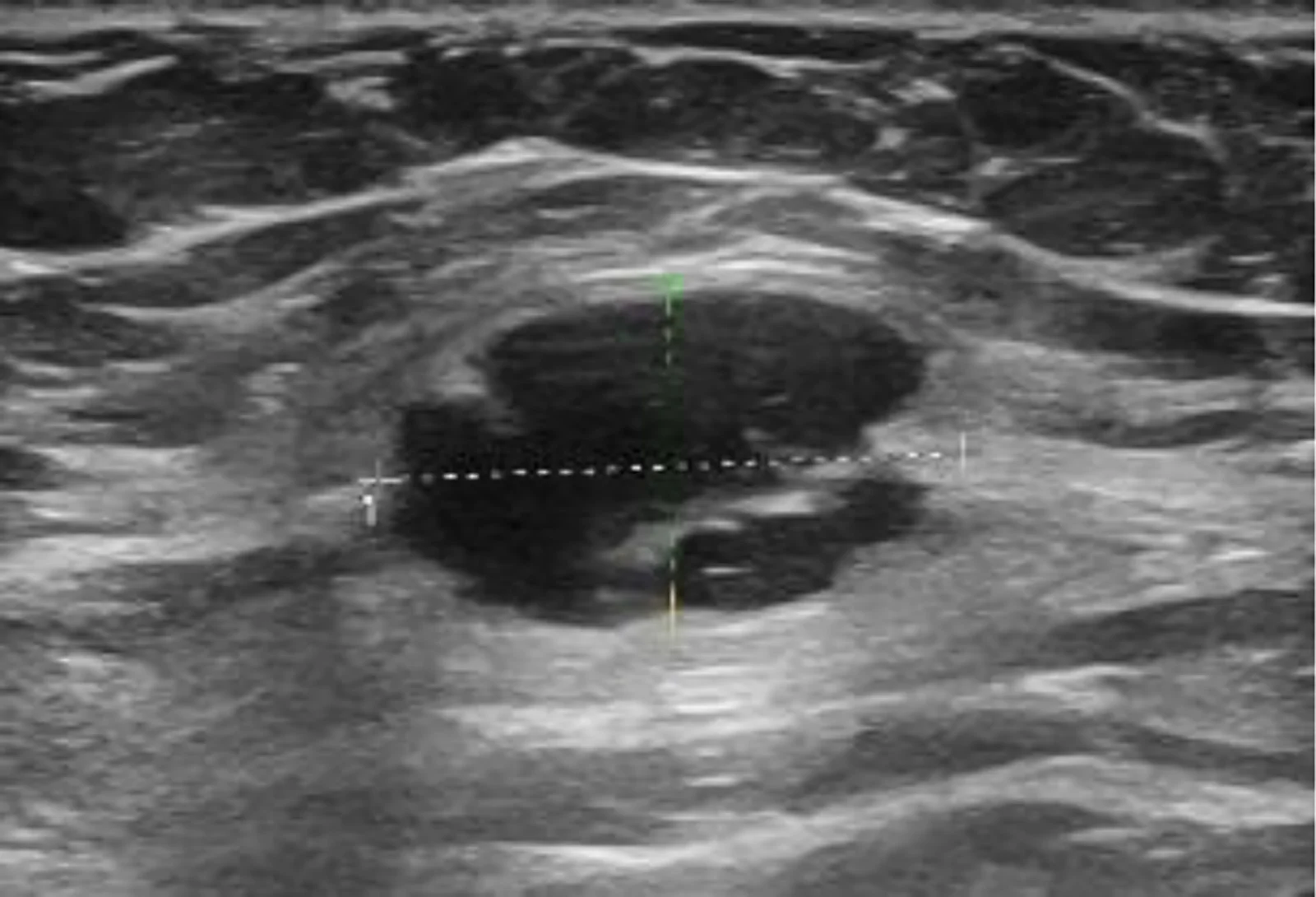
The gross morphological manifestations after complete resection of the mass.

A 67-year-old female patient was found to have a mass in the left abdominal wall one year ago, measuring approximately 4 × 2 cm. The mass was firm, tender, and exhibited limited mobility. It had enlarged significantly in recent weeks. An outpatient ultrasonography of the superficial mass revealed a solid lesion within the muscles of the left abdominal wall, prompting further inpatient evaluation (Fig. 1).

During hospitalization, the patient remained in stable condition. She had no significant past medical history other than a cesarean section performed 40 years ago, and she denied any other surgeries or traumatic injuries. After completing preoperative assessments, the patient underwent resection of a trunk muscle lesion under general anesthesia on December 26, 2024.

**Figure 2.**
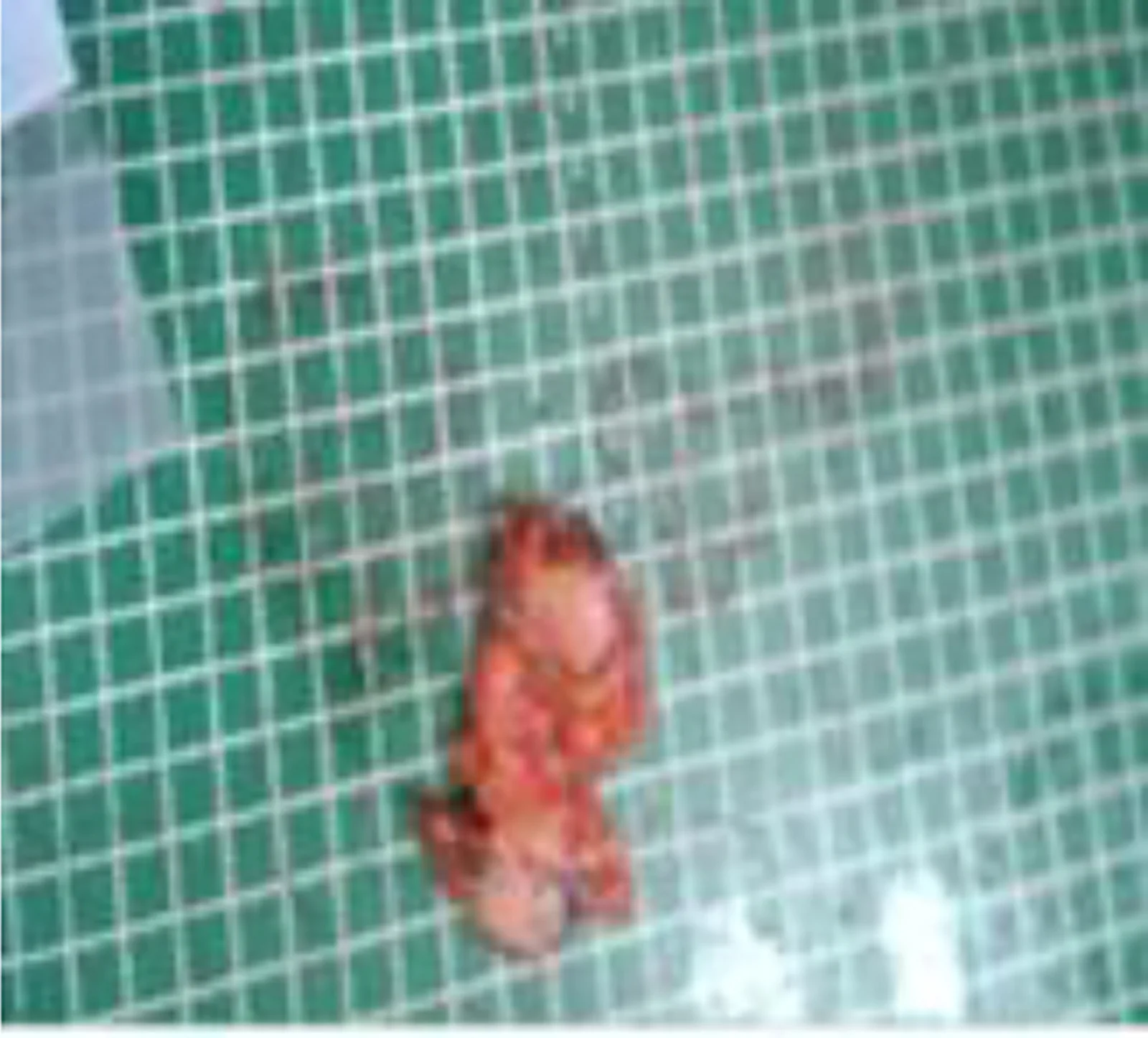
The gross morphological manifestations after complete resection of the mass.

**Figure 3.**
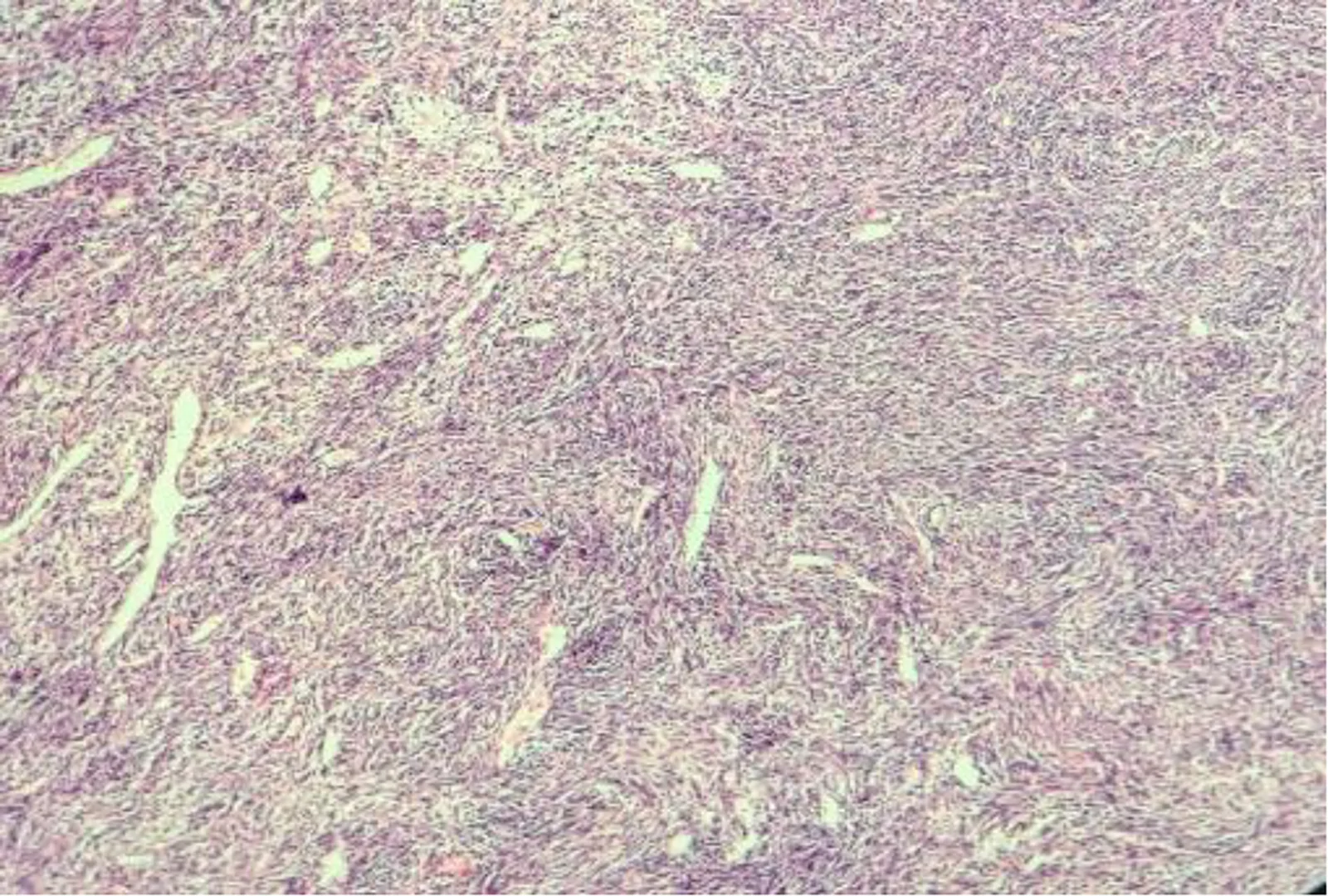
The microscopic appearance of the tumor under hematoxylin and eosin (HE) staining (100×).

Intraoperative findings identified a mass approximately 4 × 3 cm in size at the intersection of the left midclavicular line and the superior border of the costal margin (Fig. 2). The mass showed unclear boundaries and adhesion to surrounding tissues. It was excised with a 1 cm margin inferiorly. Intraoperative frozen section analysis confirmed tumor-free margins (superior, inferior, medial, lateral, and deep). Upon hematoxylin and eosin (HE) staining, a proportion of cells exhibited a spindle shape. The cell arrangement was disorganized..The tumor cells showed marked atypia and were diffusely distributed (Fig. 3). The postoperative routine pathology showed: (Muscle mass) Mesenchymal-derived malignant tumor. Based on immunohistochemistry (IHC) and fluorescence in situ hybridization (FISH) findings, the diagnosis was consistent with synovial sarcoma. No definite vascular tumor emboli or perineural invasion was identified, with a small amount of skeletal muscle observed at the periphery. All resection margins were clear.

**Figure 4.**
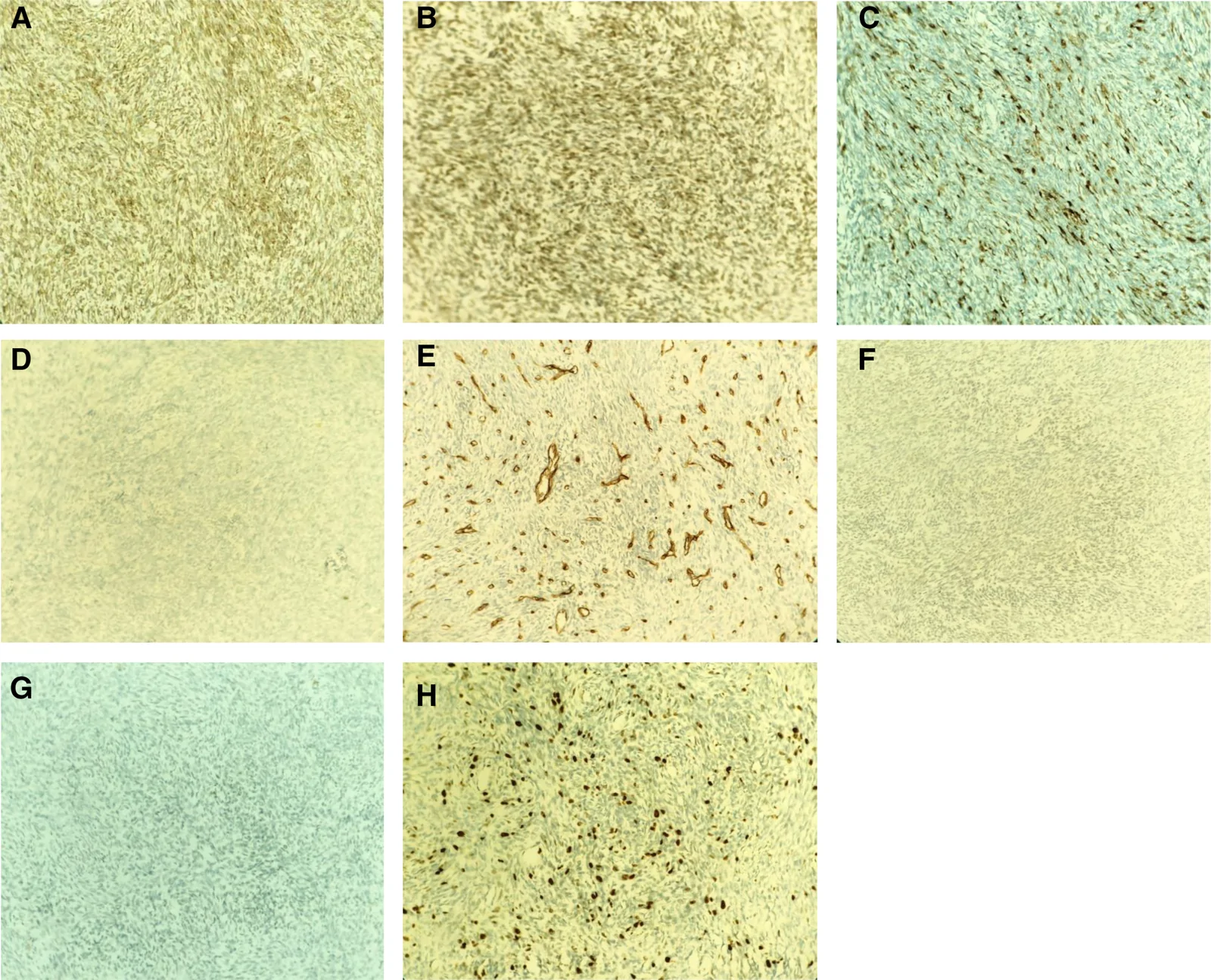
(A) A subset of cells exhibited positive immunoreactivity for BCL-2. (B) A fraction of cells showed positive staining for H3K27Me3. (C) A fraction of cells showed positive staining forP16. (D) A proportion of cells exhibited positive reactivity for cytokeratin (CK). (E) In tumors, the blood vessels showed positive expression of CD34. (F) EMA was expressed positively in a proportion of cells. (G) CD10 was expressed positively in a subset of cells. (H) In tumor cells, the positive rate of Ki-67 was 20%.

Immunohistochemical results were as follows: BCL-2(+) (Fig. 4A), H3K27Me3(+) (Fig. 4B), Calponin (focal+), P16 (focal+) (Fig. 4C), Pan-CK (scattered+) (Fig. 4D), SMA (-), CD34 (vascular+) (Fig. 4E), S-100(-), EMA (focal+) (Fig. 4F), CD99(-), PR (-), CD10 (scattered+) (Fig. 4G), Desmin(-), STAT6(-), Ki-67 (index 20%) (Fig. 4H).

**Figure 5.**
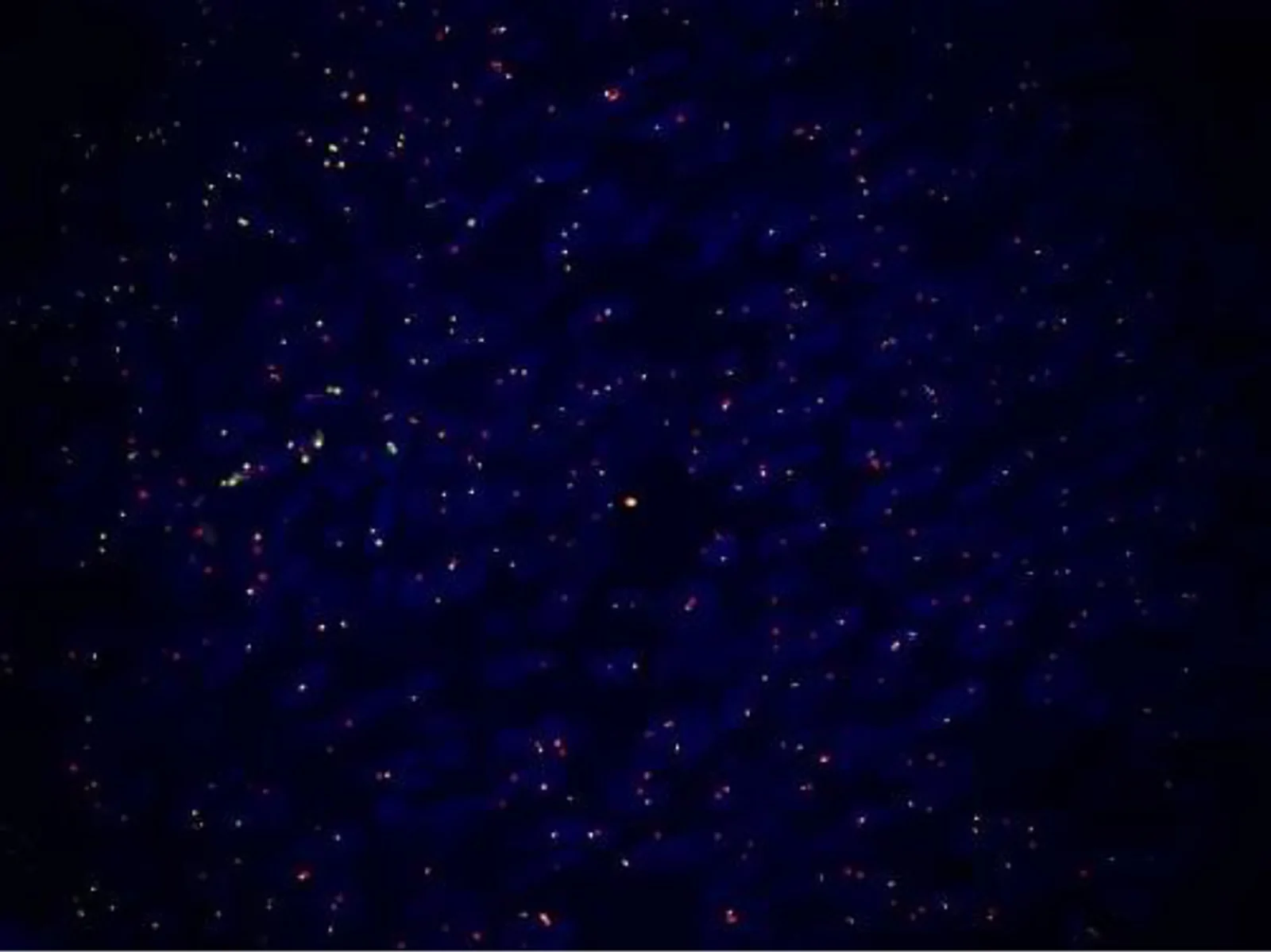
FISH (Fluorescence in situ Hybridization) gene testing revealed a positive result for breakage of GSP SS18 (SYT).

FISH analysis confirmed the presence of a SS18 (SYT) gene rearrangement. (Fig. 5).

Following surgery, the patient was referred to medical oncology for adjuvant chemotherapy. As of February 8, 2025, she has completed 4 cycles of chemotherapy. The regimen, dosed according to her body surface area, consisted of: Epirubicin 150 mg, Day 1 + Cyclophosphamide 2.5 g, Days 1–6, repeated every 3 weeks.

**Figure 6.**
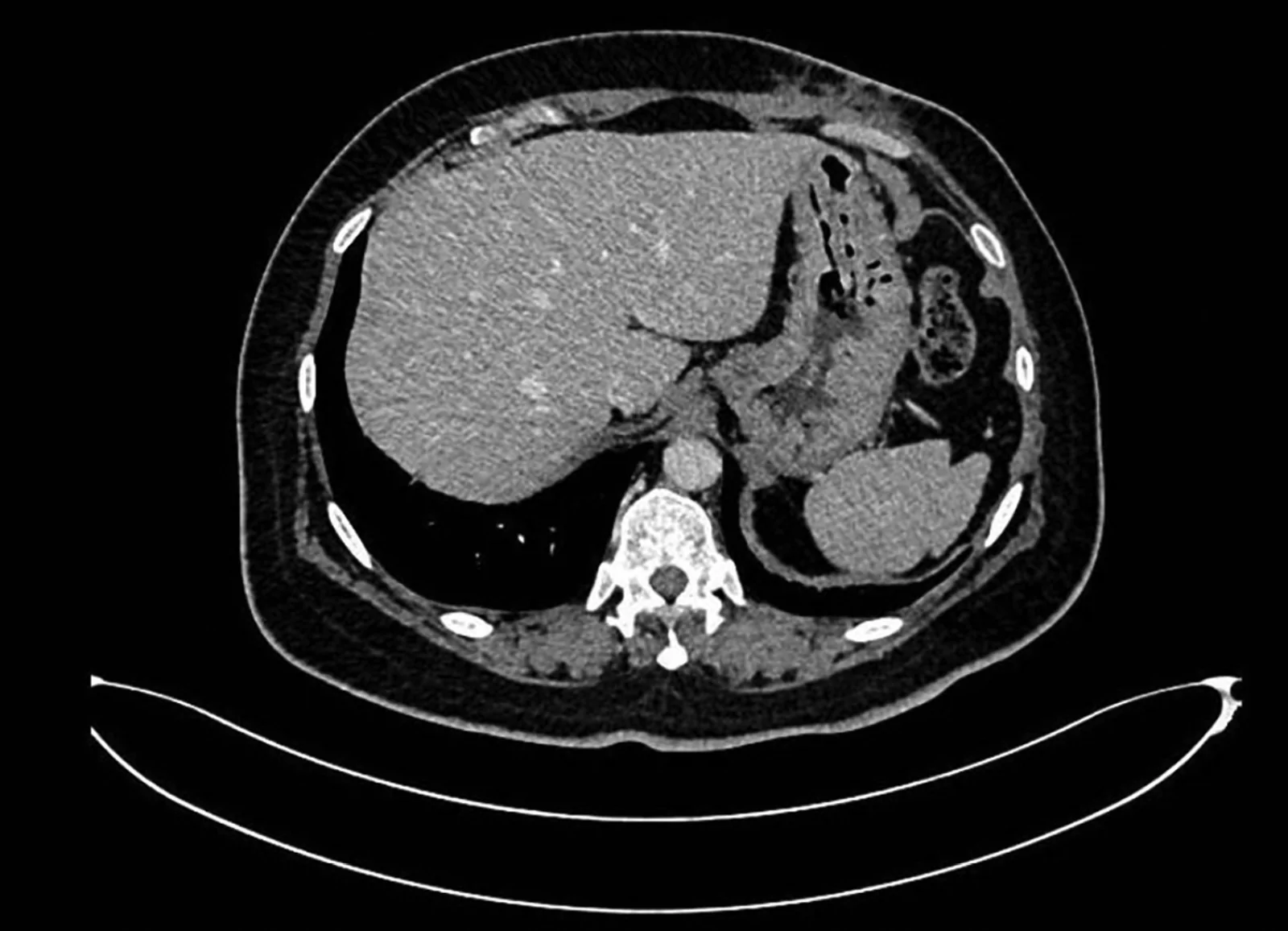
Gross appearance of the complete excision of the mass.

**Figure 7.**
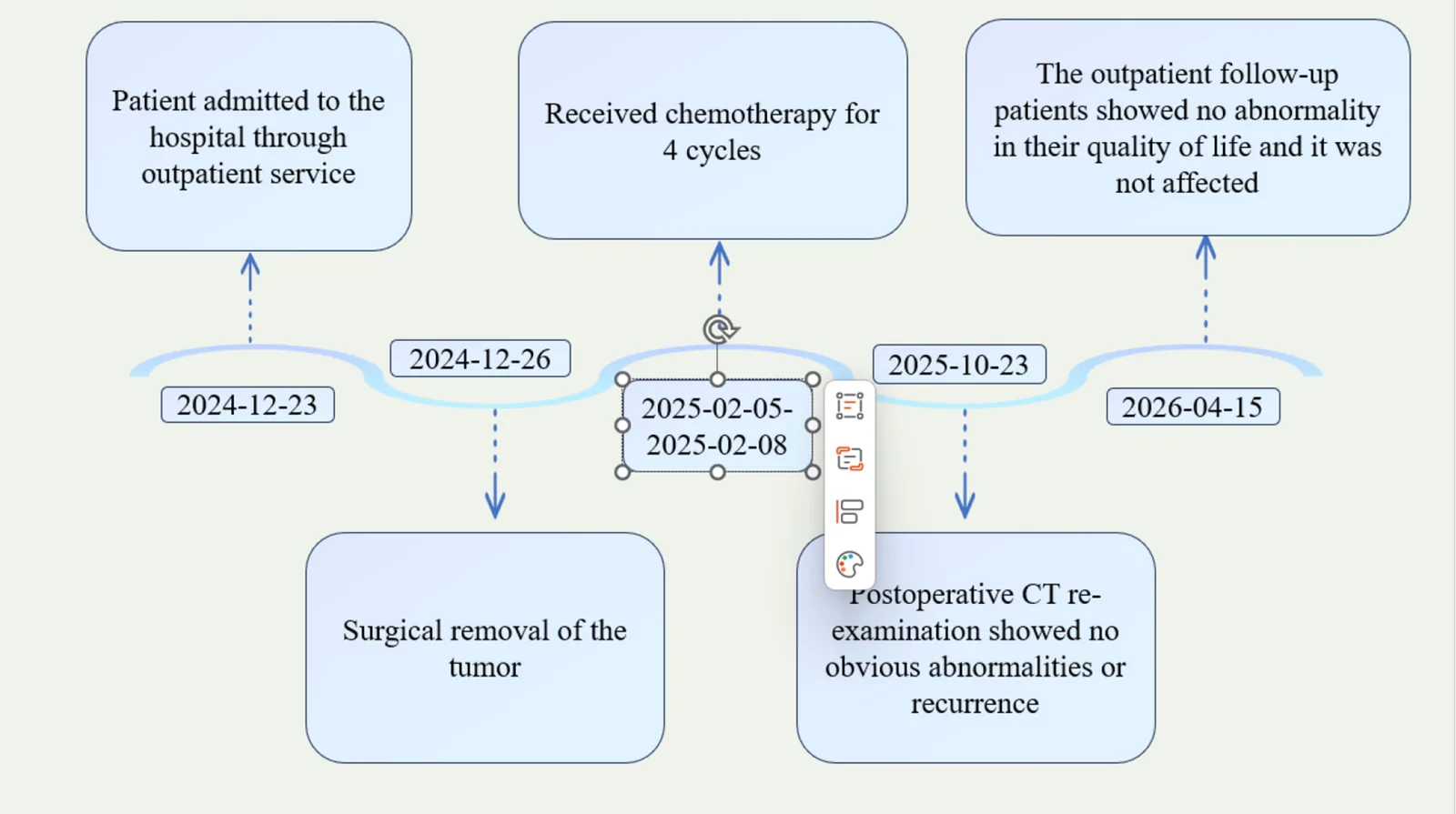
Patient-related diagnosis and treatment schedule.

A postoperative abdominal CT scan showed patchy opacities in the left anterior abdominal wall with no evidence of metastasis. (Fig. 6). The schedule for the patient’s relevant diagnosis and treatment is presented in Figure 7.

## 3. Discussion

SS, first reported in 1934, is a malignant soft tissue tumor originating from undifferentiated mesenchymal cells and accounts for approximately 5–10% of all soft tissue sarcomas. This rare and aggressive malignancy, whose etiology remains unclear, can affect individuals of any age but is most frequently observed in adolescents and young adults.

Current consensus indicates that SS does not arise from synovial cells, nor does it exhibit synovial differentiation. Instead, it is believed to originate from primitive multipotent mesenchymal cells. Histologically, SS is classified into 4 subtypes: monophasic spindle cell, monophasic epithelial, classic biphasic (the most common form), and poorly differentiated.^[3–5]^

Although SS typically occurs near large joints, it can also develop in locations unrelated to synovial tissue. Tumors arising in the chest or abdominal wall are particularly uncommon, representing only about 5% of all SS cases. Those occurring in atypical anatomical sites often lack distinctive clinical features, leading to frequent misdiagnosis prior to surgery. Definitive diagnosis in such cases relies heavily on immunohistochemical analysis and fluorescence in situ hybridization (FISH) detection of the characteristic SS18-SSX fusion gene.

### 3.1. Clinical and pathological features

Abdominal wall SS lacks distinct clinical manifestations. Most cases are incidentally detected via imaging studies such as abdominal ultrasound or CT during physical examinations. A minority of patients may experience mild tenderness. In this case, the mass was identified by ultrasound after palpation.

Due to the absence of specific morphological characteristics, IHC analysis of surgical or biopsy specimens is essential for distinguishing SS from other soft tissue sarcomas. Commonly used IHC markers for SS include CK pan, BCL-2, EMA, CD99, CK19, and TLE1.^[6]^ The IHC results for this patient were as follows:

BCL-2 (+), H3K27Me3 (+), Calponin (focal+), P16 (focal+), Pan-CK (scattered+), SMA (–), CD34 (vascular+), S-100 (–), EMA (focal+), CD99(–), PR (–), CD10 (scattered+), Desmin (–), STAT6(–), Ki-67.

Since other soft tissue malignancies such as fibrosarcoma and carcinosarcoma may show overlapping IHC profiles, fluorescence in situ hybridization (FISH) is often employed for definitive diagnosis. Most SS cases exhibit SS18 gene rearrangement, with typical red-green separation signals observed in nuclei. Molecular genetic testing is currently a critical method for differentiating SS from other sarcomas.

SS is molecularly characterized by the t(X;18) (p11.2; q11.2) translocation, which results in the expression of an aberrant SS18–SSX fusion protein. This specific genetic alteration is considered the gold standard for SS diagnosis and offers potential targets for molecular therapy.

### 3.2. Imaging manifestations and characteristics

CT Density Characteristics: Compared to abdominal muscle tissue, SS typically presents as a heterogeneous soft-tissue-density mass. Larger tumors may show internal cystic changes, necrotic low-density areas, and slightly hyperdense hemorrhagic regions, but generally no fat density. Some studies^[7]^ report calcifications, usually small patchy deposits along the tumor periphery; extensive or central calcification is rare but considered characteristic. Contrast-enhanced CT reveals significant tumor enhancement. While smaller solid lesions enhance uniformly, larger masses demonstrate obviously heterogeneous or progressive enhancement. Non-enhancing necrotic/cystic components are present, with abnormally enlarged feeding vessels noted at the tumor periphery.

MRI Signal Characteristics: The solid components of SS usually show iso- to slightly hyperintense signal on T2WI, iso- to hypointense signal on T1WI, and mild diffusion restriction (slight hyperintensity) on DWI. Larger tumors often display internal hemorrhage and cystic degeneration, leading to signal heterogeneity. Hemorrhage signal varies with time, and fluid-fluid levels may be observed due to sedimentation or intratumoral bleeding. Low-signal fibrous septa may also be present.

On T2WI, signal characteristics can be indicative: cystic/necrotic areas and fresh hemorrhage appear hyperintense, solid components show intermediate signal, while old hemorrhage, calcification, and fibrosis appear hypointense. Jones et al^[8]^ described this mixed signal pattern as the “triple signal sign,” though it is not universally present and is more common in larger masses.

SS is a malignant mesenchymal tumor prone to recurrence and metastasis, most commonly hematogenous spread to the lungs.^[9]^

### 3.3. Differential diagnosis

Solitary Fibrous Tumor: Usually larger in size; density closely correlates with collagen content. Myxoid degeneration, hemorrhage, necrosis, and cystic changes may occur; calcification is rare. Moderate progressive enhancement is typical, often with a “map-like” pattern. Internal tortuous vessels and a “pseudocapsule sign” may be present.

Rhabdomyosarcoma: Typically presents as a large, infiltrative mass with ill-defined margins. Calcification is uncommon, and adjacent bone destruction is rare. Enhancement is usually mild to moderate, most pronounced peripherally.

SS rarely occurs in the abdomen, and its clinical and imaging features (CT/MRI) often overlap with those of other soft tissue sarcomas, leading to frequent misdiagnosis. For unclear abdominal or soft tissue masses, MRI findings such as high DWI signal and enhancing internal septations with delayed contrast uptake should raise suspicion for SS. However, definitive diagnosis requires pathological biopsy with IHC marker analysis and, when necessary, FISH testing to reduce misdiagnosis of SS in rare locations.

### 3.4. Treatment and prognosis

There is no standardized treatment protocol for SS. Management typically involves a multimodal approach including complete surgical resection, chemotherapy, and radiotherapy. However, the prognosis remains poor, with a disease control rate of only about 30%.

Targeted therapy for refractory advanced soft tissue sarcomas is an active research area. Recent studies suggest that nab-paclitaxel combined with PD-1 inhibitors may improve outcomes in advanced cases.^[10]^ The SS18–SSX fusion gene may regulate immune response via the PD-1/PD-L1 axis, influencing tumor growth and invasion. Additionally, SS18–SSX promotes angiogenesis through VEGF secretion.^[11]^

Novel fusion genes such as SS18-NEDD4,^[12]^ EWSR1–SS18, and MN1–SS18^[13]^ have also been identified in SS, providing a theoretical basis for future targeted therapies and advancing precision medicine in SS treatment.

SS exhibits highly variable biological behavior, ranging from indolent to highly aggressive. Prognostic factors include clinical stage at diagnosis, tumor size, and histological grade according to the FNCLCC (French Federation Nationale des Centers de Lutte Contre le Cancer) system.

## 4. Conclusion

In conclusion, although intermuscular synovial sarcoma of the abdomen is an infrequent occurrence, its imaging features do exhibit certain distinct characteristics. Typically, it presents as a lobulated soft-tissue mass with marginal calcifications. The mass has relatively well-defined boundaries. Hemorrhage and necrosis tend to occur within the tumor, and fibrous septa can be observed. The “triple – signal sign” on magnetic resonance imaging (MRI) is characteristic. During contrast – enhanced scanning, it demonstrates marked heterogeneous enhancement. A comprehensive analysis of computed tomography (CT) and MRI findings, in conjunction with the clinical history and the results of percutaneous biopsy, can enhance preoperative diagnostic accuracy. This approach enables the determination of appropriate surgical strategies, thereby reducing the risk of secondary surgeries and metastasis. Moreover, integrating the results of postoperative immunohistochemistry and gene testing helps to formulate the ultimate comprehensive treatment plan, thereby minimizing the risks and losses borne by the patient.

## Author contributions

**Data curation:** Wen-xu Liu.

**Formal analysis:** Wen-xu Liu.

**Investigation:** Wen-xu Liu, Wen-Qian Liu, Ji-Guang Song, Ming-Hao Jia, Jia-Le Wang, Feng-Ling Li.

**Writing – original draft:** Wen-xu Liu.

**Writing – review & editing:** Wen-xu Liu, Jia-You Xu.

**Funding acquisition:** Jia-You Xu.

**Supervision:** Jia-You Xu.

**Validation:** Jia-You Xu.
